# A narrative review of new strategies for immunotherapy combination treatment in platinum-resistant ovarian cancer: mechanisms, clinical evidence, and future directions

**DOI:** 10.3389/fonc.2026.1792883

**Published:** 2026-06-22

**Authors:** Lili Zhang, Xin Ma, Xiaofeng Zhao

**Affiliations:** 1 Inner Mongolia Medical University, Department of Obstetrics and Gynecology, Affiliated Hospital of Inner Mongolia Medical University, Inner Mongolia, Hohhot, China; 2 Inner Mongolia Medical University, Department of Thyroid and Breast Surgery, Affiliated Hospital of Inner Mongolia Medical University, Inner Mongolia, Hohhot, China; 3 Department of Obstetrics and Gynaecology, Affiliated Hospital of Inner Mongolia Medical University, Inner Mongolia, Hohhot, China

**Keywords:** anti-angiogenesis, antibody-drug conjugates, biomarkers, immune combination therapy, immune microenvironment, PARP inhibitors, platinum-resistant ovarian cancer

## Abstract

Platinum-resistant ovarian cancer (PROC) is a major challenge in gynecological tumor treatment, with poor prognosis and limited chemotherapy efficacy. Immune checkpoint inhibitors as monotherapy demonstrate low response rates, underscoring the need for effective combination therapies. This review aims to describe recent advances in immune combination therapy for PROC, explain synergistic mechanisms, summarize key clinical evidence, and discuss challenges and future directions. The search of PubMed and Web of Science (January 2018–January 2026) following PRISMA guidelines identified phase II/III trials, prospective cohorts, and preclinical studies. The narrative review found that combination strategies with anti-angiogenic agents achieved objective response rates (ORR) of 24–27% and median progression-free survival (PFS) of 4–5 months. Adding PARP inhibitors yielded an ORR of 18–31% with disease control rates of 65–81%. Furthermore, the antibody-drug conjugate (ADC) mirvetuximab soravtansine as monotherapy achieved an ORR of 42.3% in folate receptor alpha (FRα)-high PROC. Finally, the phase III KEYNOTE B96 trial reported a 4.2-month overall survival benefit with pembrolizumab plus chemotherapy in the PD-L1-positive population. These regimens substantially improve efficacy compared with historical single-agent chemotherapy (ORR 10–20%, PFS 3–4 months). In conclusion, immune combination therapy offers a novel therapeutic strategy for PROC. Future efforts should explore biomarkers, optimize regimens, overcome resistance, and validate efficacy through innovative trials to achieve personalized treatment.

## 1 Introduction

Ovarian cancer is the most lethal malignancy of the female reproductive system, with more than 310,000 new cases and 200,000 deaths per year worldwide (1). Due to its insidious early symptoms approximately 70% of patients are diagnosed with advanced disease, defined as International Federation of Gynecology and Obstetrics, (FIGO) stage III/IV (2). Despite the high efficacy of initial treatment (tumor cytoreduction followed by platinum-based chemotherapy), up to 80% of patients with advanced-stage disease ultimately relapse (3). Recurrent platinum-resistant ovarian cancer (PROC), defined as disease progression within 6 months following the completion of the final platinum-based chemotherapy, accounts for 25-30% of recurrence cases (4). Patients with PROC have a poor prognosis, with a median overall survival (OS) of typically 12–18 months and represent a significant challenge for clinical care (5). Currently, the standard treatment options for PROC are limited and are largely restricted to non-platinum-based single-agent chemotherapies administered weekly, such as polyethylene glycolated liposomal doxorubicin (PLD), topotecan, gemcitabine (GEM), and paclitaxel (6). However, the objective response rate (ORR) of these regimens is generally low (10-20%), with a median progression-free survival (PFS) of only 3–4 months, and these regimens are frequently associated with significant toxicity that severely compromises patients’ quality of life (7). Therefore, the development of novel, highly effective and low-toxicity therapeutic strategies is required. Immunotherapy, especially immune checkpoint inhibitors (ICIs) targeting programmed death protein-1/programmed death protein ligand-1 (PD-1/PD-L1), has achieved revolutionary success in a wide range of solid tumors such as melanoma and non-small cell lung cancer (8). However, in ovarian cancer, the efficacy of monotherapy with ICIs has been less promising. The KEYNOTE-100 study, a large Phase II clinical trial, showed that the ORR of pembrolizumab in advanced recurrent ovarian cancer was only 8% (combined positive score [CPS] ≥1) and 9.7% (CPS ≥10) (9). Similarly, the JAVELIN Ovarian 200 study demonstrated that avelumab monotherapy or avelumab in combination with PLD did not significantly improve PFS or OS (10). This therapeutic resistance is primarily driven by the tendency of ovarian cancers, particularly PROC, to exhibit an “immuno-cold tumor” phenotype, which is characterized by a lack of T-cell infiltration, defective antigen presentation and a highly immunosuppressive tumor microenvironment (TME) (11). The “cold” tumor microenvironment is not static but can be remodeled by diverse therapeutic interventions. Therefore, the current focus of research and development has shifted from ICI monotherapy to mechanism-based combination strategies. These strategies aim to convert “immuno-cold” tumors into “immuno-hot” tumors, thereby reversing primary resistance to ICIs (12). To address this gap, this narrative review aims to provide a systematic and mechanistic synthesis focusing specifically on immunotherapy combinations for PROC. We aim to: (1) comprehensively analyze how each combination strategy mechanistically counteracts specific immunosuppressive pathways within the PROC TME; (2) synthesize and compare the latest clinical evidence (2018-2026) across these strategies; and (3) evaluate persistent challenges and future directions in biomarker-driven personalized therapy. This focused analysis aims to move beyond a descriptive inventory of therapeutic options and propose a rationale-driven framework for developing and personalizing immune-combination therapies in this refractory setting. Unlike previous reviews, this narrative review not only summarizes the evidence, but also focuses on analyzing the mechanistic rationale linking each strategy to specific TME defects in PROC, with an emphasis on emerging strategies (such as ADCs and bispecific antibodies) and future directions in biomarker-driven personalized therapy. Several recent reviews have provided comprehensive overviews of ovarian cancer management (2), general frameworks for tumor immunophenotypes and combination immunotherapy (12), and the evolution of precision medicine in this field (94). However, a comprehensive and systematic review of the mechanisms, latest clinical evidence, and comparative challenges of immunotherapy combinations for the most refractory PROC subtype remains unavailable. The present narrative review aims to fill this gap by offering such an analysis.

## 2 Methods

**Figure 1:**
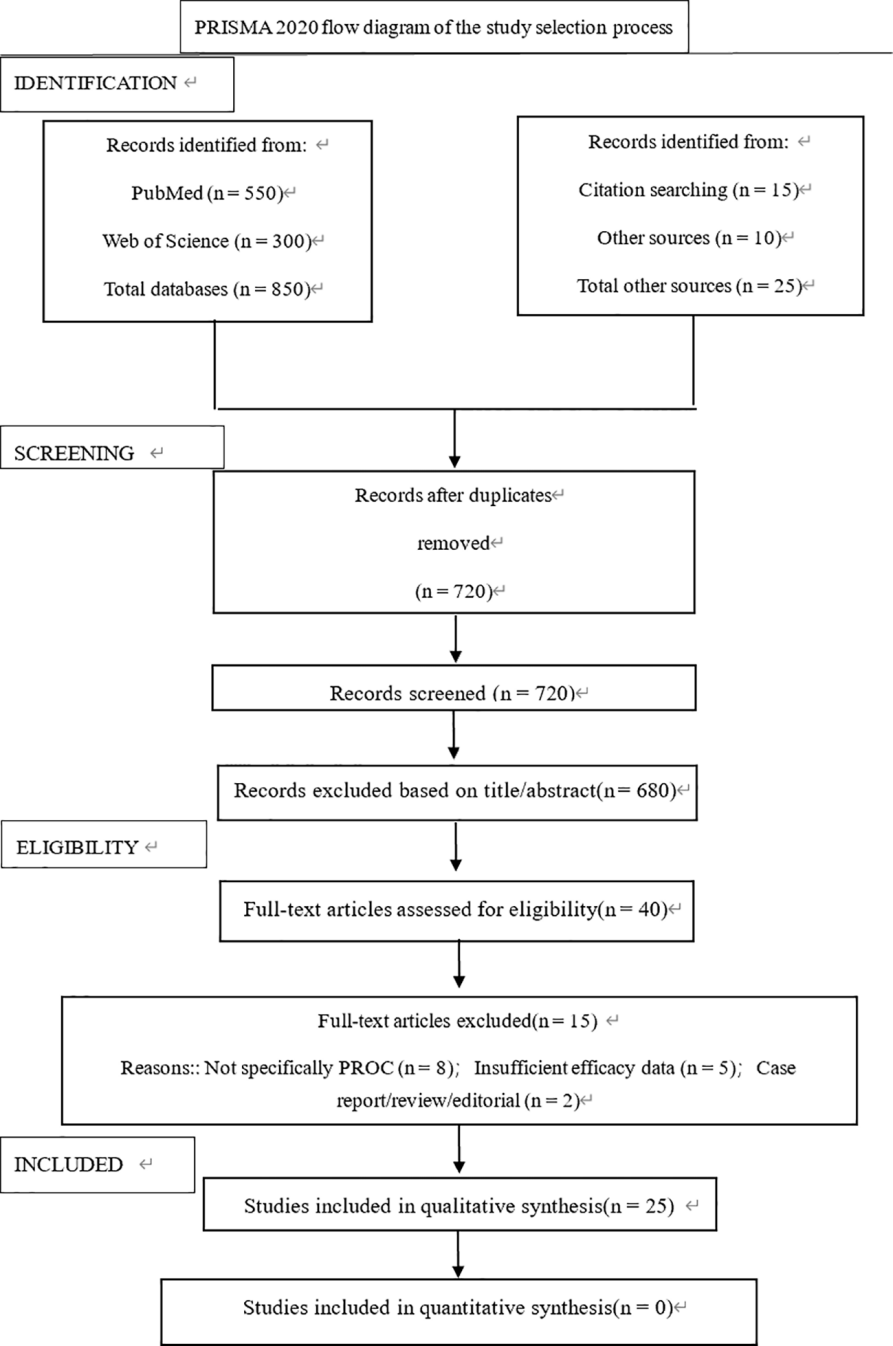
PRISMA flow diagram of study selection process.

A systematic literature search was performed in PubMed and Web of Science for studies published between January 1, 2018 and January 1, 2026. The search strategy incorporated keywords including “platinum-resistant ovarian cancer”, “immunotherapy combination”, “immune checkpoint inhibitor”, “PARP inhibitor”, “antibody-drug conjugate” and “bispecific antibody”. Eligibility was restricted to peer-reviewed articles published in English. The reference lists of retrieved articles were additionally screened for additional relevant studies. Studies were included if they: (1) enrolled patients with platinum-resistant ovarian cancer (PROC); (2) evaluated immunotherapy-based combination regimens (e.g., with anti-angiogenic agents, PARP inhibitors, chemotherapy, ADCs, or bispecific antibodies); (3) reported efficacy outcomes (ORR, PFS, OS) or safety data; (4) represented phase II/III clinical trials, prospective cohort studies, or preclinical studies providing mechanistic insights; and (5) were published as peer-reviewed articles. Studies were excluded if they: did not specifically address PROC or combination immunotherapy, were case reports, editorials, commentaries, duplicate publications, conference abstracts, clinical trial registry entries, or had insufficient data. Three independent reviewers screened the titles and abstracts, then assessed the full texts of potentially eligible studies against the inclusion criteria. Disagreements were resolved by discussion or consultation with a third reviewer. The study selection process is summarized in a PRISMA flow diagram (**Figure 1**). For included clinical trials, risk of bias was assessed using the Cochrane Risk of Bias tool (for RCTs) or the MINORS tool (for non-randomized studies), whereas preclinical studies were not formally assessed due to their inherent heterogeneity. Data, including first author, year, study design, patient characteristics, treatment regimen, efficacy outcomes, and adverse events, were extracted using a standardized form. This review is a narrative synthesis of the literature, as a meta-analysis was not feasible due to substantial heterogeneity. Consequently, a narrative synthesis was conducted, with studies grouped by combination strategy.

## 3 Immune microenvironment in PROC

The marked therapeutic resistance of platinum-resistant ovarian cancer (PROC) is inextricably linked to its distinctively adapted tumor microenvironment (TME). The TME is not a passive backdrop but an active pathophysiological driver, shaped by the selective pressures of platinum therapy.

### 3.1 Cellular architecture: a physical barrier and a suppressive network

At the cellular level, the PROC TME is characterized by a pronounced imbalance: a paucity of effector cells and a dense infiltration of immunosuppressive populations. A hallmark of PROC is the markedly reduced infiltration of cytotoxic CD8^+^ T cells and helper CD4^+^ T cells into the tumor parenchyma (13). This “T-cell exclusion” is not merely a passive absence but an active process driven by cancer-associated fibroblasts (CAFs). CAFs, activated by factors such as TGF-β (transforming growth factor-beta), form a dense, fibrotic stroma by secreting abundant extracellular matrix (ECM) proteins, creating a physical barrier. Furthermore, CAFs secrete chemokines such as CXCL12, which effectively “sequesters” T cells within the peritumoral stroma, preventing their physical interaction with cancer cells and creating a functional “immune-excluded” phenotype (14, 15). Within the excluded stroma and the tumor itself, a potent network of suppressor cells actively suppresses any residual effector function (16). Highly enriched in ovarian cancer, Tregs (regulatory T cells) directly suppress effector T cells through contact-dependent inhibition and the secretion of immunosuppressive cytokines such as IL-10 (interleukin-10) and TGF-β (17). This heterogeneous population inhibits T cells through multiple mechanisms, including the depletion of essential amino acids and the generation of reactive oxygen species (18). Unlike anti-tumor M1 counterparts, M2-TAMs promote angiogenesis, tissue remodeling, and immunosuppression by secreting IL-10, TGF-β, and VEGF, thereby reinforcing the protumorigenic environment (19).

### 3.2 Molecular mediators: the cytokine and chemokine milieu

The cellular architecture is governed by a complex milieu of soluble factors that foster an environment hostile to immune effector function. Beyond its canonical role in promoting aberrant angiogenesis, VEGF-A is a direct immunosuppressor. It inhibits dendritic cell (DC) maturation, thereby impeding the initiation of adaptive immunity. It also promotes the recruitment and expansion of Tregs and myeloid-derived suppressor cells (MDSCs) while upregulating inhibitory checkpoint molecules such as PD-1 on the surface of infiltrating T cells (20). The resulting structurally and functionally aberrant vasculature also exacerbates hypoxia and elevates interstitial fluid pressure, thereby further impairing immune cell trafficking (21). TGF-β and IL-10 represent two of the most potent immunosuppressive cytokines in the PROC TME. TGF-β directly inhibits the cytotoxic activity of CD8^+^ T cells and natural killer (NK) cells while simultaneously promoting CAF activation and ECM deposition (22). IL-10, secreted by multiple cell types including Tregs and M2-polarized tumor-associated macrophages (M2-TAMs), impairs the antigen-presenting function of DCs and macrophages, thereby attenuating the T-cell response (23). Metabolic stress and cell death in the TME lead to the release of ATP, which is rapidly hydrolyzed to adenosine by the ectonucleotidases CD39 and CD73, expressed on both tumor and immune cells. High concentrations of adenosine bind to the A2A receptor on T cells and NK cells, potently suppressing their proliferation, cytokine production, and cytotoxicity (24).

### 3.3 Metabolic dysregulation and the vicious cycle of the “Cold” state

These cellular and molecular features converge to establish a self-reinforcing “cold” tumor phenotype, driven by profound metabolic dysregulation (25). Rapid tumor growth outstrips its blood supply, creating chronic hypoxia. This stabilizes hypoxia-inducible factor-1α (HIF-1α), which transcriptionally upregulates VEGF and TGF-β, thereby promoting M2-TAM polarization, CAF activation, and further angiogenesis, ultimately establishing a feedback loop that perpetuates T-cell exclusion (26). The overexpression of indoleamine 2,3-dioxygenase 1 (IDO1) by tumor cells and MDSCs depletes local tryptophan and generates kynurenine. Tryptophan starvation inhibits CD8^+^ T cell proliferation, while kynurenine promotes the differentiation of naïve CD4^+^ T cells into immunosuppressive Tregs, effectively depriving effector cells of essential nutrients and simultaneously fueling the suppressor network (27). The Warburg effect results in high lactate production, acidifying the TME. This acidic environment directly suppresses the cytotoxicity of CD8^+^ T cells and NK cells via GPR81 signaling and enhances the suppressive function of MDSCs via the upregulation of molecules like arginase 1 (26).

### 3.4 Risk of bias assessment

The methodological quality of the included studies varied according to study design. The Cochrane Risk of Bias 2.0 tool was used to assess the major RCTs identified in this review. **Table 1** summarizes the risk-of-bias assessments for the key RCTs. Most RCTs demonstrated low risk of bias overall, primarily attributable to well-described randomization methods, adequate allocation concealment, and complete reporting of primary outcomes. For example, the TOPACIO/KEYNOTE-162 trial, which evaluated niraparib in combination with pembrolizumab, demonstrated a low risk of bias across the randomization, missing data, and outcome measurement domains, although some concerns remained regarding the selective reporting of secondary endpoints. The IMagyn050 trial (atezolizumab with bevacizumab and chemotherapy) also exhibited low risk of bias in most domains, with an overall rating of ‘some concerns’ primarily arising from the open-label design, which could introduce detection bias for non-blinded subjective outcomes such as pain or symptom assessment.

**Table 2:** Tumor microenvironment characteristics and their impact on immune response.

Microenvironment feature	Major effector cells/molecules	Impact on immune response	Potential targeting strategies	Representative drugs/targets
Insufficient immune cell infiltration (26, 51, 73)	Low infiltration of CD8^+^ T cells	Lack of anti-tumor immune effector cells	Recruit T cells	Chemotherapy, radiotherapy, STING agonists and OX40 agonists
Enrichment of immunosuppressive cells (14–16, 90)	Tregs, MDSCs and M2-TAMs	Suppress effector T cell function	Deplete or inhibit immunosuppressive cells	Anti-CSF-1R, anti-CCR4, anti-CD25 and PI3Kγ inhibitors
Immune checkpoint expression (8, 9, 98)	PD-1/PD-L1 and CTLA-4	T cell exhaustion	Immune checkpoint blockade	Pembrolizumab, nivolumab and ipilimumab
Immunosuppressive factors (18–20, 92)	VEGF, TGF-β and IL-10	Inhibit DC maturation and T cell activation	Neutralize suppressive factors or block their pathways	Bevacizumab (anti-VEGF), TGF-β trap and anti-IL-10R
Physical barriers (17, 22, 89)	Fibrotic stroma and CAFs	Impede T cell infiltration	Target CAFs or anti-fibrosis	Anti-FAP, PEGPH20 (hyaluronidase) and LOXL2 inhibitors
Metabolic microenvironment (21, 26–28, 91)	Adenosine, IDO and hypoxia	Suppress T cell metabolism and function	Reverse metabolic suppression	Anti-CD73, anti-A2AR, IDO inhibitors and HIF-1α inhibitors
Imbalanced cytokine network (73, 79, 92)	Lack of co-stimulatory signals and excess of inhibitory cytokines	Ineffective T cell activation	Provide co-stimulation/block inhibitory cytokines	IL-2, IL-12, 4-1BB agonists, OX40 agonists, TGF-β Trap and anti-IL-10R

This complex immunosuppressive landscape explains the low response rate of ICI monotherapy and points to the direction of combination therapy: a multi-pronged approach is necessary to break immunosuppression at multiple points in order to effectively activate anti-tumor immunity.

HIF-1α, hypoxia-inducible factor-1α; Tregs, regulatory T cells; MDSCs, myeloid-derived suppressor cells; TAMs, tumor-associated macrophages; PD-1, programmed death protein-1; PD-L1, programmed death protein ligand-1; VEGF, vascular endothelial growth factor; IL, interleukin; CAFs, cancer-associated fibroblasts.

For non-randomized prospective cohort studies and phase I/II single-arm trials, the ROBINS-I tool was used. The key limitations identified across these studies included potential confounding due to the absence of a concurrent control group (in single-arm trials), selection bias arising from non-consecutive patient enrollment, and incomplete reporting of baseline prognostic factors that could influence treatment outcomes. Specific concerns were identified for several single-arm phase II studies in which the lack of comparator arms limited the ability to control for time-varying confounding (e.g., subsequent lines of therapy after progression). The majority of these non-randomized studies were rated as having moderate risk of bias overall, indicating that the studies were sound for non-randomized designs but could not be considered comparable to well-performed RCTs.

We utilized the MINORS tool for non-comparative observational studies and retrospective analyses. The most frequent methodological limitations included the lack of prospective data collection, the failure to report prospective calculation of study size, loss to follow-up exceeding 5%, and the absence of blinding in endpoint assessment. However, most studies clearly stated their aims, included consecutive patients, and used endpoints appropriate to the research question. The MINORS total scores ranged from 11 to 21 out of a maximum of 24, indicating overall fair to moderate methodological quality. Regarding the application of findings in the narrative interpretation, studies rated as having a high or critical risk of bias were not excluded from the review but were interpreted with caution, with their findings discussed separately as exploratory evidence rather than conclusive support for the efficacy of a given regimen. Substantial heterogeneity in study designs and patient populations precluded quantitative sensitivity analyses. Consequently, the narrative discussion in the following sections explicitly highlights which reported findings are derived from studies with lower risk of bias to ensure that clinical recommendations are grounded in the most reliable available evidence (**Table 2**).

**Table 1:** Summary of risk of bias assessment of key clinical trials.

Trial name (phase)	Study design	Assessment tool	Overall RoB rating	Key rationale
JAVELIN Ovarian 200 (III)	RCT (three-arm)	RoB 2.0	Low	Rigorous randomization and allocation concealment; independent central review of endpoints; low attrition
IMagyn050 (III)	RCT (double-blind)	RoB 2.0	Low	Double-blind design with placebo control; independent endpoint review; low missing data
SORAYA (III)	Phase III single-arm (accelerated approval)	ROBINS-I	Low	Single-arm by design (FDA registration); high-quality data monitoring; independent central review
BRIGHT study (II)	Umbrella trial (multi-arm)	RoB 2.0	Low	Biomarker-driven randomization; pre-specified statistical analysis plan
TOPACIO/KEYNOTE-162 (II)	RCT (open-label)	RoB 2.0	Some concerns	Lack of blinding; no pre-specified analysis plan for some secondary endpoints
MEDIOLA (II) ovarian cohort	Phase II basket (single-arm)	ROBINS-I	Moderate	No control arm; confounding risk; baseline imbalances not fully adjusted
China basket study (II)	Phase II single-arm	ROBINS-I	Moderate	Single-arm design; potential selection bias; post-hoc subgroup analyses
Lenvatinib + Toripalimab (II)	Phase II single-arm	ROBINS-I	Moderate	Limited sample size; no control arm; well-defined PFS as primary endpoint
InnovaTV 205 (I/II)	Phase I/II (dose-expansion)	ROBINS-I	Moderate	Dose-finding phase; small sample size in PROC cohort; heterogeneity in prior treatments
KEYNOTE-100 (II)	RCT (open-label)	RoB 2.0	Some concerns	Open-label design; outcome assessors not blinded; participant-reported outcomes may be affected

+, activation.

−, inhibition.

OC, ovarian cancer; ORR, objective response rate; PLD, polyethylene glycolated liposomal doxorubicin; PFS, progression-free survival; OS, overall survival; PROC, platinum-resistant ovarian cancer; FRα, folate receptor α; TV, tisotumab vedotin.

## 4 Immunological combination therapy strategies

**Figure 2:**
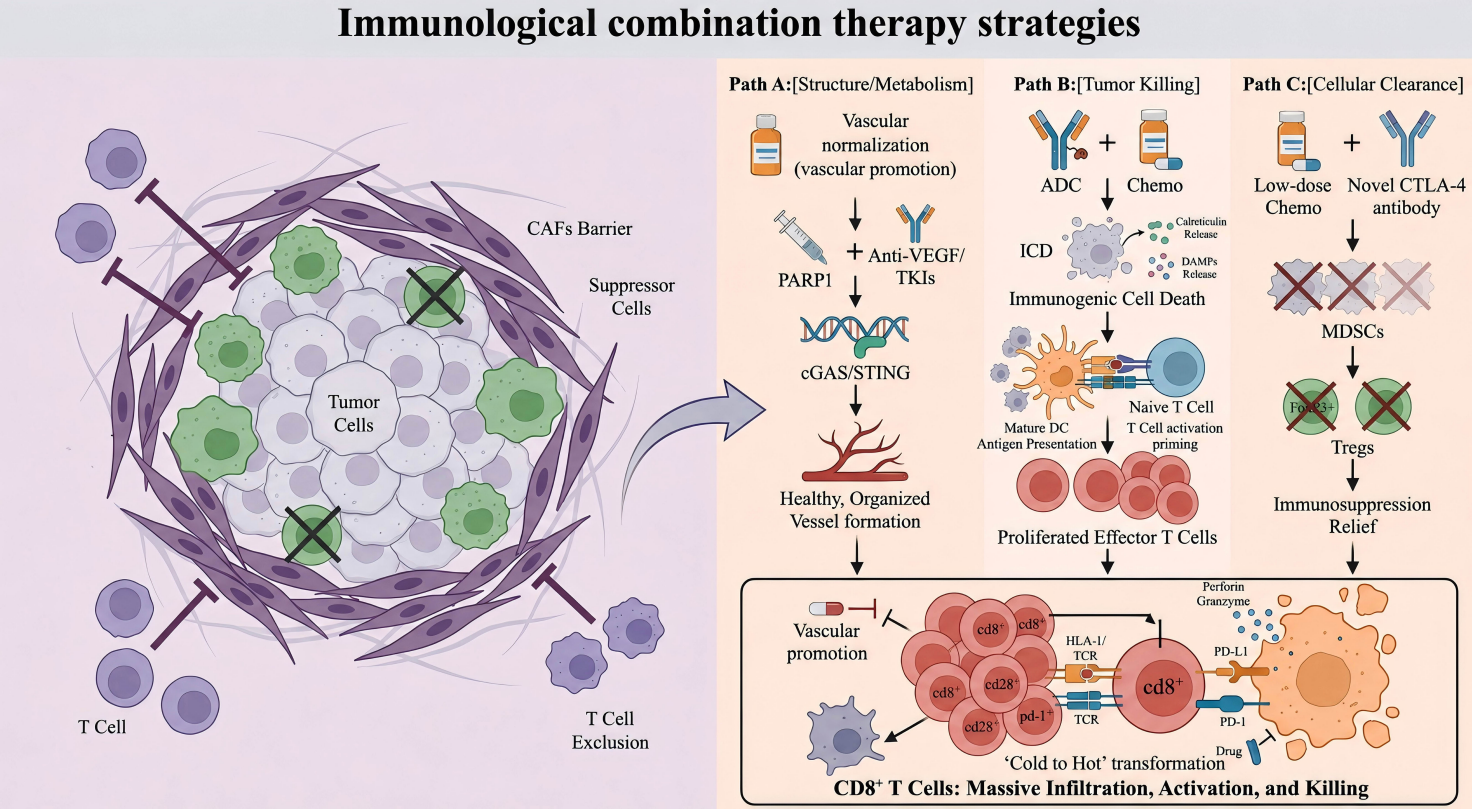
Three major synergistic pathways by which immunotherapy combined treatment remodels the platinum-resistant ovarian cancer ‘cold’ tumor microenvironment. This image illustrates the mechanisms of three main combination treatment strategies: Path A (structural and metabolic regulation): PARP inhibitors combined with anti-VEGF/VEGFR drugs improve immune cell infiltration by regulating tumor metabolism and vascular structure; Path B (tumor killing and immune activation): Antibody-drug conjugates (ADCs) combined with chemotherapy directly kill tumor cells, induce immunogenic cell death (ICD), and release antigens and DAMPs; Path C (removal of suppressive cells): By eliminating myeloid-derived suppressor cells (MDSCs) and regulatory T cells (Tregs), immune suppression is relieved.

Clinical data from key Phase II studies, as detailed below and summarized in **Table 3**, indicate that the combination of ICIs with anti-angiogenic agents yields an objective response rate (ORR) of 20-40% and a progression-free survival (PFS) of 4–6 months in patients with PROC. These historical data show significantly better results compared to traditional single-agent chemotherapy. Angiogenesis and immunosuppression form a vicious cycle in the TME. VEGF-A, as a core molecule, not only promotes the formation of structurally aberrant and dysfunctional tumor vasculature, leading to tissue hypoxia and elevated interstitial fluid pressure, but also directly suppresses the immune response (20). The combination of anti-angiogenic drugs (such as the VEGF antibody bevacizumab and VEGFR [vascular endothelial growth factor receptor] tyrosine kinase inhibitors [TKIs]) and ICIs has a solid theoretical basis. The synergistic mechanisms include vascular normalization, which not only remodels tumor vasculature to improve perfusion and alleviate hypoxia (thereby facilitating drug and T cell delivery) (21), but also reverses VEGF-mediated immunosuppression by restoring dendritic cell maturation and antigen presentation. Furthermore, anti-angiogenic therapy reduces checkpoint molecule expression on T cells, restoring their effector function (28), while normalized vessels upregulate adhesion molecules to promote T cell attachment and extravasation (28).Several clinical studies have explored the value of this combination strategy in PROC. The immunotherapeutic combination involving VEGF antibodies was demonstrated in the Phase III IMagyn050/GOG 3015/ENGOT-OV39 study, which evaluated the efficacy of atezolizumab in combination with paclitaxel, carboplatin, and bevacizumab in the first-line treatment of newly diagnosed advanced ovarian cancer. Although the final results did not meet the primary endpoint of PFS (30), a trend toward improvement was observed in the PD-L1-positive population, thereby providing a rationale for subsequent studies. In the PROC field, several phase II studies have shown promise. For example, a Chinese phase II basket study evaluated the efficacy of camrelizumab (anti-PD-1) in combination with famitinib (multi-targeted TKI) in patients with PROC. Among the 37 patients enrolled, the ORR was 24.3%, the disease control rate (DCR) was 54.1%, the median PFS was 4.1 months, and the median OS was 18.9 months. Regarding safety, the profile was generally manageable despite a high incidence of grade 3 or higher treatment-related adverse events (TRAEs; 81.1%) (29) (**Figure 2**).

**Table 3:** Comparison of major immunotherapy combination strategies for platinum-resistant ovarian cancer.

Joint strategy	Core coordination mechanism	Key clinical evidence (ORR/PFS)	Main advantages	Main limitations and challenges
ICI anti-angiogenic drugs (33)	Vessel normalization reverses VEGF-mediated immunosuppression	Carrelizumab + famitinib; ORR, 24.3%; mPFS, 4.1 months	Rich clinical experience, able to quickly improve the tumor microenvironment	Toxicities such as hypertension and proteinuria may accumulate; the therapeutic effect may be temporary
ICI PARPi (44)	Activate the cGAS/STING pathway to increase tumor immunogenicity	Niraparib + Pembrolizumab; ORR, 18%; DCR, 65%	Strong theoretical synergy, especially for HRD tumors	High risk of hematologic toxicity and irAEs; Phase III trial did not meet its endpoints
ICI chemotherapy (57)	Induce immunogenic cell death and eliminate suppressive cells	Relacorilant with albumin-bound paclitaxel; ORR, 36.9%; mPFS, 6.54 months	Familiar with chemotherapy frameworks, some regimens can modulate immunity	Traditional chemotherapy is toxic, and the immune system may be suppressed by chemotherapy
ICI ADCs (35, 58, 63)	Precision killing releases antigens, and the load induces ICD	Mirvetuximab soravtansine (monotherapy); ORR, 42.3% (high FRα expression)	Efficient and precise, overcoming the heterogeneity of the bystander effect	Unique adverse reactions such as ocular toxicity and neurotoxicity
ICI dual antibodies/new drugs (74, 75)	Multi-target blockade or providing co-stimulatory signals	Gotistobart pembrolizumab; ORR, ~26%	The mechanism is innovative and may improve the therapeutic window	Mostly early data, long-term safety unknown

PARPi, PARP inhibitor; ORR, objective response rate; PFS, progression-free survival; VEGF, vascular endothelial growth factor; ICI, immune checkpoint inhibitor; DCR, disease control rate; HRD, homologous recombination repair-deficient; irAEs; immune-related adverse events; ADCs, antibody-drug conjugates; ICD, immunogenic cell death; FRα, folate receptor α.

Lenvatinib, a multi-targeted TKI, has attracted attention for its potent immunomodulatory effects. A multicenter Phase II study investigated low-dose lenvatinib (14 mg/day) in combination with Toripalimab in patients with PROC after multiple lines of therapy. In the cohort of 33 patients, the study demonstrated an ORR of 27% and a DCR of 54%, alongside a median PFS of 5.0 months and a median OS of 13.3 months (30, 31). Notably, the regimen exhibited a manageable safety profile, with grade 3 TRAEs observed in only 39% of patients and no grade 4 or 5 events (30).

This “chemo-immuno-antiangiogenic” paradigm combining chemotherapy, anti-vascular agents, and immunotherapy may maximize efficacy through synergistic mechanisms, but the superimposed toxicity must be closely monitored. Superimposed toxicity refers to the phenomenon in which combining multiple therapeutic agents results in toxicities that are not merely additive but potentially synergistic, leading to a higher incidence, greater severity, or an entirely new adverse event profile compared to any single agent or doublet combination. In the context of this triple regimen, hematologic toxicity from chemotherapy can be compounded by the bone marrow effects of anti-angiogenic TKIs and the immune-related cytopenias occasionally seen with ICIs. Vascular adverse events, such as hypertension and proteinuria, represent well-established class effects of anti-VEGF therapy (e.g., bevacizumab). The concomitant administration of an ICI, which can independently cause rare but serious vascular events such as vasculitis or myocarditis, creates an uncertain and potentially dangerous overlapping risk profile.

Immune-related adverse events (irAEs) associated with ICIs may overlap with and clinically mimic toxicities induced by chemotherapy or anti-angiogenic agents. Therefore, although this case report demonstrates that favorable outcomes with manageable toxicity are achievable in carefully selected patients, it does not mitigate the broader risk of superimposed toxicity within a larger, more heterogeneous population. The BRIGHT study and similar trials are crucial for systematically evaluating whether the enhanced efficacy of this triple combination outweighs the substantially elevated risk of complex, overlapping toxicities. Close monitoring, proactive management, and potential dose modifications are paramount for the safe implementation of this therapeutic approach.

Combining immunotherapy with anti-angiogenic agents has a strong theoretical rationale for reversing the immunosuppressive microenvironment and enhancing T-cell infiltration. Furthermore, several Phase II trials have demonstrated objective response rates (ORRs) surpassing those of traditional chemotherapy. Despite these advantages demonstrated in Phase II trials, clinical application still faces significant challenges. First, the enhanced efficacy may be accompanied by significantly elevated toxicities, such as hypertension, proteinuria and hand-foot syndrome, necessitating careful dose management and patient monitoring (32). Compared to combining immunotherapy with PARP (poly (ADP-ribose) polymerase) inhibitors or chemotherapy, this strategy offers the advantage of rapidly modulating the tumor’s physical and chemical barriers, thereby facilitating immune cell infiltration. Furthermore, some regimens (such as low-dose lenvatinib-based combinations) have shown manageable safety profiles (30). Several phase II trials have evaluated this combination in PROC. The camrelizumab plus famitinib basket study (n=37) reported an ORR of 24.3%, a median PFS of 4.1 months, and a median OS of 18.9 months, albeit with high-grade TRAEs in 81.1% (29). A multicenter phase II trial of low-dose lenvatinib (14 mg/day) plus toripalimab (n=33) achieved an ORR of 27%, a median PFS of 5.0 months, and a more manageable safety profile (grade ≥ 3 TRAEs 39%, with no grade 4 or 5 events) (30). In contrast, the phase III IMagyn050 trial evaluating atezolizumab added to paclitaxel, carboplatin and bevacizumab in first-line advanced ovarian cancer did not meet its primary PFS endpoint, although a trend toward benefit was seen in PD-L1-positive subgroups (33). Taken together, ICI–anti-angiogenic combinations can approximately double the ORR (20–27%) compared with historical single-agent non-platinum chemotherapy (ORR 10–20%), but optimal patient selection and toxicity management remain critical. The negative result of the phase III IMagyn050 trial in the first-line setting does not contradict the positive signals seen in phase II studies of anti-angiogenic plus ICI combinations in PROC. The former enrolled treatment-naïve patients with immunologically cold tumors, where triple therapy added toxicity without efficacy; the latter included heavily pretreated, platinum-resistant patients whose tumors may have acquired neoantigens and partial immune priming after multiple lines of chemotherapy, making them more likely to show a signal. Therefore, positive phase II results in PROC should not be extrapolated to first-line practice, nor be taken as proof of general efficacy of this strategy. Most of the “positive” findings in PROC derive from single-arm, small-sample studies after extensive prior therapy, lacking independent phase III validation. However, its limitations include the potentially limited durability of efficacy and the lack of robust predictive biomarkers akin to homologous recombination repair-deficiency (HRD) for PARP inhibitors. Future research should investigate the association between angiogenesis-related molecular features (such as VEGF/VEGFR expression profiles and circulating endothelial cells) and therapeutic response and optimize the timing and sequencing of combination therapies through adaptive clinical trial designs to strike an optimal balance between efficacy and toxicity.

The combination of immunotherapy and PARP inhibitors represents a promising therapeutic strategy. Clinical evidence indicates that the combination of immunotherapy and PARP inhibitors holds significant therapeutic promise for a subset of patients with PROC. Key Phase II studies, such as TOPACIO/KEYNOTE-162, have demonstrated an objective response rate (ORR) of approximately 18% and a disease control rate (DCR) of nearly 65% for the combination therapy. These findings indicate efficacy in patient populations beyond those harboring BRCA (breast cancer susceptibility gene) mutations. These studies are discussed in detail in the following sections.

PARP inhibitors eliminate HRD tumor cells through a ‘synthetic lethality’ effect, while also exhibiting potent immunomodulatory effects, which are theoretically synergistic with ICIs (34). i) Enhancement of tumor immunogenicity: The PARP inhibitor-induced DNA damage response increases genomic instability, leading to elevated tumor mutational burden (TMB) and neoantigen generation (35). Additionally, DNA fragments accumulated in the cytoplasm can activate the cyclic GMP-AMP synthase–stimulator of interferon genes (cGAS-STING) pathway and induce type I interferon production, which in turn promotes DC maturation, antigen cross-presentation and T cell recruitment (36); ii) Regulation of PD-L1 expression: Several studies have shown that PARP inhibitors (such as olaparib and niraparib) upregulate PD-L1 expression on tumor cells and immune cells, which may make tumors more sensitive to PD-1/PD-L1 blockade (37); and iii) Modulation of immune cell function: PARP inhibitors may directly modulate Treg activity. It has been shown that PARP-1 deletion or inhibition can weaken the inhibitory function of Tregs, thereby enhancing anti-tumor immunity (38). Clinical evidence for this can be found with the TOPACIO/KEYNOTE-162 study. This Phase II basket trial evaluated the efficacy of niraparib combined with pembrolizumab for treating PROC, irrespective of BRCA status. Among the 62 patients with PROC evaluable for efficacy, the ORR was 18%, and the DCR reached 65%. Notably, the ORR still reached 11% in BRCA wild-type patients, suggesting that this combination regimen may overcome resistance to PARP inhibitors (39). Furthermore, the MEDIOLA study, a Phase II basket trial, investigated the efficacy of olaparib plus durvalumab across various solid tumors. The ovarian cancer cohort (including platinum-sensitive and platinum-resistant patients) showed an ORR of 31% and a DCR of 81% in 32 evaluable patients. Response was observed in both BRCA mutated and non-mutated patients (40). These findings contrast sharply with earlier views that considered PARP inhibitors solely as ‘synthetic lethal’ agents. Instead, they reveal the central role of PARP inhibitors in modulating tumor immunogenicity, thereby providing a theoretical foundation for overcoming the immune “cold” phenotype of PROC “cold” phenotype of PROC. Updated survival data demonstrated a median OS of 17.8 months for the entire MEDIOLA cohort (40, 41). The BRIGHT study demonstrated a precision medicine strategy. This umbrella trial was designed specifically for a cohort of patients with PROC carrying a BRCA mutation, treated with pamiparib (a PARP inhibitor) in combination with bevacizumab. This biomarker (BRCA mutation)-based precision therapy strategy, which aimed to maximize the efficacy of PARP inhibitors and combine them with anti-angiogenic immunomodulatory effects, represents the future direction of individualized therapy (42). Despite the encouraging results of these Phase II studies, large Phase III trials evaluating combinations of immunotherapy and PARP inhibitors in the first-line maintenance setting—such as DUO-O (durvalumab + bevacizumab ± olaparib) and FIRST (durvalumab ± olaparib)—failed to meet their primary efficacy endpoints in the overall population and were associated with higher rates of toxicity. In DUO-O, the addition of olaparib did not significantly improve investigator-assessed progression-free survival (PFS) (hazard ratio [HR] 0.87, 95% CI 0.74–1.03; p=0.11), and the incidence of grade ≥3 adverse events was higher in the olaparib arm (57% vs. 49%) (43). Similarly, FIRST demonstrated no PFS benefit and a higher incidence of serious adverse events with combination therapy (42). The FIRST/ENGOT-OV44 trial (NCT03602859) demonstrated that the addition of dostarlimab to first-line platinum-based chemotherapy followed by niraparib maintenance yielded a statistically significant improvement in PFS compared with niraparib alone (HR 0.85, 95% CI 0.73–0.99; P < 0.035), although no significant difference in overall survival was observed (42). These findings suggest that while adding immunotherapy to PARP inhibitor-based regimens may enhance efficacy in the frontline setting for ovarian cancer, the magnitude of this benefit and its impact on overall survival warrant further investigation. The safety profiles were generally consistent with the known toxicities of each agent.

Several factors may account for the discordance between the promising Phase II results in PROC and the unfavorable Phase III results in frontline settings:

1. Patient population heterogeneity – Phase II studies enrolled heavily pretreated PROC patients with accumulated DNA damage, which may have potentiated the immunomodulatory effects of PARP inhibitors. In contrast, first-line patients may have intrinsically “immune-cold” tumors less responsive to immunotherapy.
2. Inadequate biomarker selection – Phase II data demonstrated efficacy even in BRCA wild-type patients, but Phase III trials lacking enrichment for homologous recombination deficiency (HRD) or immune-related biomarkers may have diluted the treatment effect.
3. Toxicity burden limiting efficacy – Overlapping hematologic toxicity may have led to dose reductions or early discontinuations, thereby compromising the potential synergistic effect.
4. Differential effects in frontline vs. recurrent settings – PARP inhibitor-mediated immune activation (e.g., cGAS-STING pathway) may be more pronounced after multiple lines of therapy, in the presence of pre-existing immune recognition.

These findings underscore critical lessons for the future development of ICI and PARP inhibitor combinations in PROC: patient selection must be guided by multidimensional biomarkers (e.g., HRD status, immune gene signatures, tumor-infiltrating lymphocytes), overlapping toxicities necessitate careful management, and the sequencing and timing of these combination regimens require optimization. Instead of relying on extrapolations from frontline studies, PROC-specific trials should adopt biomarker-driven enrichment strategies and maintain realistic expectations regarding effect sizes. The phase II TOPACIO/KEYNOTE-162 trial of niraparib plus pembrolizumab in PROC (n=62) demonstrated an ORR of 18% and DCR of 65%, with responses observed even in BRCA wild-type patients (ORR 11%) (39). The MEDIOLA phase II basket trial of olaparib plus durvalumab in an ovarian cancer cohort (including both platinum-sensitive and platinum-resistant patients) reported an ORR of 31% and DCR of 81% in 32 evaluable patients, with a median OS of 17.8 months (40). However, subsequent phase III trials in the frontline setting failed to show PFS benefit in unselected populations and were associated with increased hematologic toxicity (42, 43). The discordance between promising phase II results and negative phase III outcomes strongly suggests that ICI–PARPi combinations should be restricted to biomarker-selected patients, particularly those with HRD-positive or STING-pathway-activated tumors, rather than applied unselectively.

While the combination of immunotherapy and PARP inhibitors is theoretically compelling due to mechanisms such as cGAS/STING pathway activation, and although it has demonstrated efficacy in select patients with PROC in Phase II trials, Phase III clinical trials (e.g., DUO-O and FIRST) have failed to demonstrate a benefit in the overall population. This indicates that broad, indiscriminate application of this strategy may unnecessarily increase the risk of hematologic toxicity and irAEs without providing a significant survival benefit. In contrast to the combination of immunotherapy and anti-angiogenic agents, the advantage of this strategy lies in its synergy, which is rooted in the inherent DNA damage repair defects of tumor cells, potentially inducing more durable immune memory. However, a notable limitation is that this strategy requires a higher baseline level of tumor immunogenicity and carries a risk of overlapping toxicity. The efficacy of this strategy is highly dependent on the genomic characteristics (such as HRD status) of the tumor and the baseline immune activity of the TME. Future research directions should prioritize shifting from a broad population approach to precise selection, relying on multidimensional biomarkers, including HRD, BRCA mutations, STING pathway activity, and tumor-infiltrating lymphocyte (TIL) density, to precisely delineate the population most likely to benefit.

Immunological combination chemotherapy. The clinical development of immunochemotherapy is predicated on the principle that certain chemotherapeutics can enhance, rather than merely suppress, anti-tumor immunity, primarily through the induction of immunogenic cell death (ICD). While numerous large-scale Phase III trials are currently underway, select rigorously designed regimens have already demonstrated promising results. The ongoing ROSELLA Phase III trial (NCT05257408) is evaluating the glucocorticoid receptor modulator relacorilant in combination with nab-paclitaxel; this approach represents an innovative strategy to mitigate chemotherapy-induced immunosuppression (44). This section will summarize the relevant clinical evidence, commencing with the mechanisms of action.

Previously, chemotherapy was regarded as universally immunosuppressive due to its myelosuppressive effects. However, a growing body of evidence indicates that specific chemotherapeutic agents, when administered at particular doses and schedules, can enhance anti-tumor immunity through several mechanisms (45). Immunogenic Cell Death (ICD): Agents such as anthracyclines, oxaliplatin, and taxanes (e.g., paclitaxel) induce a distinct form of apoptosis that exposes and/or releases damage-associated molecular patterns (DAMPs). Key examples include the surface exposure of calreticulin and the extracellular release of ATP and high mobility group protein B1 (HMGB1). DAMPs recruit and activate dendritic cells (DCs), promoting the phagocytosis of tumor cells and the subsequent presentation of tumor antigens to T cells, thereby effectively acting as an *in situ* vaccine (46). Elimination of Immunosuppressive Cells: Certain chemotherapeutics can selectively deplete immunosuppressive populations. For instance, gemcitabine (GEM) and low-dose cyclophosphamide have been shown to reduce the number and suppressive function of myeloid-derived suppressor cells (MDSCs) and regulatory T cells (Tregs), thereby relieving a key brake on effector T cells (45). Reduction of Tumor Burden and Increased Antigen Exposure: Chemotherapy-induced cytoreduction can alleviate tumor-induced systemic immunosuppression (e.g., by reducing cytokine sinks) and facilitate the expansion of newly activated T-cell clones. The substantial release of tumor-associated antigens (TAAs) resulting from tumor cell lysis further amplifies the nascent immune response (47, 48).

### 4.1 The unique role of gemcitabine in PROC: clinical evidence and mechanistic rationale

Gemcitabine (GEM), a nucleoside analog, holds a distinctive position in the treatment of PROC. Its clinical efficacy was demonstrated in a multicenter retrospective cohort study (n=130), which reported that GEM, administered as a non-platinum monotherapy, yielded a median overall survival (OS) of 15.2 months—a significant improvement over the 11.0 months observed in patients receiving other single-agent regimens (49). The rationale for combining GEM with immunotherapy in PROC is twofold and mechanistically compelling. Enhanced Cytotoxicity in PROC Subtypes: The unique metabolic features of certain ovarian cancer subtypes, particularly clear cell ovarian carcinoma (OCCC), may render them particularly susceptible to GEM. In OCCC, hyperactivation of glycolysis and the pentose phosphate pathway, driven by hypoxia-inducible factors, promotes the synthesis of nucleotide precursors. This metabolic milieu facilitates the incorporation of the active triphosphate metabolite of GEM into tumor cell DNA, thereby potentially augmenting its cytotoxic efficacy (50). Potent Immunomodulation via MDSC Depletion: Beyond its direct cytotoxic activity, GEM is well-established for its capacity to selectively deplete MDSCs (45). Clinical Relevance of MDSC Depletion in PROC: As noted above, GEM effectively reduces the MDSC burden in this setting. In the heavily immunosuppressive PROC microenvironment, where MDSCs are a major driver of T-cell dysfunction (51), this depletion represents a critical immune-sensitizing step. By depleting MDSCs, GEM can ‘prepare’ the TME for subsequent immune activation, making the combination of GEM with an ICI a highly attractive strategy to both reduce tumor burden and relieve a key layer of immunosuppression.

### 4.2 Future directions: optimizing chemo-immunotherapy

Combining immunotherapy with chemotherapy offers substantial promise, but its success depends critically on the careful selection of both the chemotherapeutic agent and the dosing regimen. The ideal partner for ICI should be an agent that optimally induces ICD while exerting minimal impact on proliferating immune effector cells. The ROSELLA Phase III study, which evaluated the GR modulator relacorilant in combination with nab-paclitaxel, illustrates a novel approach to attenuating the immunosuppressive side effects often associated with standard chemotherapy (44). For GEM, future research should focus on prospectively validating its synergistic potential with ICIs in biomarker-defined subgroups (e.g., OCCC or patients with high baseline MDSC levels) and optimize the sequence and dosing to harness its MDSC-depleting effect without causing lymphotoxicity. The landscape of chemo-immunotherapy in PROC has recently been transformed by the phase III KEYNOTE-B96 trial, which evaluated pembrolizumab added to paclitaxel (with or without bevacizumab) in 643 patients with PROC (10, 12). In the PD-L1–positive population (CPS ≥ 1), the pembrolizumab regimen achieved a 4.2-month improvement in median overall survival — the longest OS reported to date in this setting — and met its primary endpoint of PFS in both the PD-L1–positive subgroup and the overall population (12). The phase III ROSELLA trial of relacorilant plus nab-paclitaxel also showed promising activity (ORR 36.9%, median PFS 6.54 months) (52). Conversely, the AGO-OVAR 2.29 trial of atezolizumab plus non-platinum chemotherapy did not improve PFS or OS, indicating that the chemotherapy backbone and the inclusion of bevacizumab are critical determinants of success (16). Collectively, these data establish pembrolizumab-based chemo-immunotherapy as the first ICI-containing regimen with a proven overall survival benefit in PROC, representing a new standard of care.

Immune-combined antibody-drug conjugates. ADCs as a monotherapy have shown notable antitumor activity in PROC. For example, folate receptor α (FRα)-targeted drugs have demonstrated an ORR of >40% in biomarker-selected populations. Their combination with ICIs aims to enhance antitumor immunity, and early clinical data suggest potential synergistic effects. This section will review the relevant clinical progress, commencing with the mechanism of action. ADCs are composed of monoclonal antibodies targeting TAAs, a cytotoxic payload, and a linker connecting these components, and are termed “biological missiles” (53). Synergistic mechanisms of combined ADC and ICI therapy include: i) Precise targeting and efficient killing: ADCs specifically bind to TAAs on the surface of tumor cells via their antibody moiety and release potent cytotoxic drugs after internalization to effectively kill the target cells, a process that does not rely on traditional immune recognition (54); ii) induction of ICD: Some ADC payloads (such as MMAE and DM1) can induce ICD by releasing DAMPs and tumor antigens, activating DCs and T cells, effectively acting as a targeted “*in situ* vaccination” (55); iii) bystander effect: Certain linker-payload combinations (such as a cleavable linker paired with a hydrophobic payload) can enable toxic molecules to penetrate the cell membrane and kill neighboring tumor cells exhibiting low antigen expression or those lacking antigen expression, overcoming tumor heterogeneity (56); modulation of the immune microenvironment: ADC-mediated tumor cell killing can alter the TME, such as reducing immunosuppressive cells and upregulating the expression of inflammatory factors, thus establishing a microenvironment conducive to ICI efficacy (57). Building on these mechanistic rationales, several ADCs have been evaluated clinically in PROC, either as monotherapy or in combination with ICIs.

Mirvetuximab soravtansine (MIRV) is an antibody-drug conjugate (ADC) that targets FRα and is conjugated with the maytansinoid derivative DM4. Its Phase III SORAYA trial confirmed single-agent activity in patients with FRα-overexpressing PROC, with an ORR of 42.3% (58). Consequently, it has received accelerated approval from the FDA. Several trials (such as the GLORIOSA Phase III trial) are currently exploring the efficacy of MIRV in combination with ICIs (such as pembrolizumab), with preliminary data showing higher response rates (59, 60). Beyond FRα, other targets have also been explored. Tisotumab vedotin (TV) is an antibody-drug conjugate (ADC) directed against tissue factor (TF) and conjugated to MMAE. The InnovaTV 205 study included an ovarian cancer cohort and showed that TV, both as monotherapy and in combination with pembrolizumab or carboplatin, exhibited anti-tumor activity (61). Notably, among patients with PROC, TV monotherapy yielded an ORR of 22% (62).

Other ADC targets under investigation in ovarian cancer include mesothelin (MSLN), sodium-dependent phosphotransporter protein 2b (NaPi2b) and human trophoblast cell surface antigen 2 (TROP2) (63). In addition to FRα and TF, a growing list of ADC targets is under active investigation for PROC. For example, the MSLN-targeting ADC anetumab ravtansine has shown antitumor activity in a Phase I study of patients with advanced solid tumors, including those with ovarian cancer with high MSLN expression. In the ovarian cancer expansion cohort, the drug demonstrated an ORR of 37.5% (3/8) among patients with platinum-resistant disease, highlighting its potential in this challenging subtype (64). The combination of this agent with chemotherapy is currently being explored in PROC (NCT02751918) (65). Upifitamab rilsodotin (XMT-1535) targets NaPi2b, and early studies have demonstrated efficacy in ovarian cancer (66). The combination of ADCs with immunotherapy represents a promising synergy between precision medicine and immune-based strategies. Future challenges lie in optimizing target selection, managing overlapping toxicities (such as ocular toxicity and peripheral neurotoxicity) (66, 67) and exploring optimal biomarkers to identify patients who are most likely to benefit. In addition to FRα and TF, ADCs targeting other molecules offer novel avenues for PROC treatment. ADCs targeting MSLN, such as anetumab ravtansine, have shown antitumor activity in a Phase I study in ovarian cancer patients with high MSLN expression, and their combination with ICIs is currently being explored (trial no. NCT01439152) (64). Its combination with other agents is under active investigation; notably, a previously cited study reported an objective response rate of 37.5% (65). The TROP2-targeting ADC sacituzumab govitecan has been approved for the treatment of metastatic triple-negative breast cancer and locally advanced or metastatic urothelial carcinoma. In a Phase I/II basket trial (IMMU-132-01) for advanced ovarian cancer, it achieved an ORR of 33%, with a median duration of response of 8.1 months (68). Clinical investigations into its combination with pembrolizumab for PROC are currently ongoing. Furthermore, the NaPi2b-targeted ADC upifitamab rilsodotin (XMT-1535) has demonstrated encouraging activity in patients with PROC within a Phase I expansion cohort (66). In the PROC subset of the UPLIFT study, the confirmed ORR was 34.4% (11/32), with a median duration of response of 7.4 months, results that support further development in this indication (66). Combining these emerging ADCs with immunotherapy underscores the potential of future strategies that integrate precise targeting with immune activation. ADC monotherapy has already demonstrated robust activity in biomarker-selected PROC. The phase III SORAYA trial of mirvetuximab soravtansine (MIRV) in FRα-high PROC reported an ORR of 42.3%, leading to accelerated FDA approval (59). The phase Ib/II GLORIOSA trial is currently evaluating MIRV plus pembrolizumab, with preliminary data suggesting higher response rates than monotherapy (61). Tisotumab vedotin (TV), a tissue factor-targeting ADC, showed an ORR of 22% as monotherapy in PROC (InnovaTV 205) and is being studied in combination with pembrolizumab (62, 63). Other ADCs targeting MSLN, TROP 2, and NaPi2b have also demonstrated clinical activity (65–67, 69). The high ORRs of ADCs in biomarker-selected populations provide a strong rationale for combining them with ICIs to convert cytotoxic killing into durable adaptive immunity. The major clinical challenges include overlapping toxicities (e.g., ocular events and peripheral neuropathy) and the necessity for validated companion diagnostics to guide patient selection.

Novel immunization combination strategies. Novel combination strategies such as bispecific antibodies, agonist antibodies, and next-generation immune checkpoint inhibitors (ICIs) are still in the early stages of clinical exploration. Preliminary data show that certain novel regimens (such as new CTLA-4 antibodies combined with PD-1 inhibitors) have achieved an ORR of approximately 25% in patients with PROC and suggest an improved safety profile compared with that of traditional combinations. This section will present the latest clinical evidence for these emerging strategies. Building upon these approaches, more advanced immune conjugation strategies are emerging. In addition to established combination therapies, several next-generation immunotherapy strategies—including bispecific antibodies, agonist antibodies, and next-generation immune checkpoint inhibitors with refined pharmacological properties—are emerging for PROC. Although most remain in early clinical development, preliminary data have shown encouraging activity.

Bispecific antibodies. Bispecific antibodies can bind to two different antigenic epitopes at the same time, and there are two main forms in PROC: i) Immune cell engagers (T cell engagers): Such as bispecific antibodies targeting MSLN and CD3, which recruit T cells into close proximity with tumor cells, bypassing TCR specificity, and activate them to kill the target cells (69). Drugs such as AMG 701 (targeting BCMA and CD3) are under investigation; and ii) dual ICIs: Drugs such as cadonilimab (AK104), which block both PD-1 and CTLA-4, theoretically activating T cells more efficiently, while possibly reducing toxicity by altering Fc region properties (70). Other target combinations such as PD-1/T cell immunoreceptor with Ig and ITIM domains (TIGIT) and PD-1/lymphocyte activation gene-3 (LAG-3) are also in development (71). The bispecific antibody targeting both PD-1 and CTLA-4, cadonilimab (AK104), has shown significant efficacy in a phase II clinical trial for patients with previously treated cervical cancer (NCT03852251) and was approved in China in June 2022 for this indication following the results of the pivotal phase II study (NCT04380805) (70). Its application in the PROC field is currently being evaluated in an ongoing Phase II study (trial no. NCT05430906) (72), with comprehensive efficacy and safety results pending. Notably, in cervical cancer, cadonilimab monotherapy achieved an objective response rate (ORR) of 33.0% and a manageable safety profile in patients with recurrent or metastatic disease. Given its similar immunomodulatory mechanisms, cadonilimab holds promise for activity in PROC, which awaits validation in ongoing trials (70). Another class of novel immunotherapies aims to directly activate T cells rather than block inhibitory signals.

Agonistic antibodies. Agonistic antibodies against co-stimulatory molecules (such as OX40 (CD134 (tumor necrosis factor receptor superfamily member 4)), GITR (glucocorticoid-induced TNFR-related protein), ICOS (inducible T-cell costimulator) and 4-1BB (CD137)) are designed to provide direct activation signals to enhance T cell proliferation, activation and persistence (73). In combination with ICIs, they can provide “signal 2” co-stimulation to overcome barriers to T cell activation. Despite early mixed clinical results, optimizing dosing regimens and administration schedules remains a research priority. The novel CTLA-4 inhibitor gotistobart (BNT316/ONC-392) is a pH-sensitive anti-CTLA-4 monoclonal antibody. Its dissociation from CTLA-4 in the acidic environment of the lysosome facilitates the recirculation of CTLA-4 to the cell surface rather than its degradation. This unique design aims to deplete regulatory T cells (Tregs) more selectively within the tumor microenvironment while preserving peripheral Tregs—particularly those in lymph nodes—that are crucial for maintaining systemic immune homeostasis. This approach aims to enhance anti-tumor efficacy by relieving intratumoral immunosuppression while potentially mitigating the severe immune-related adverse events (irAEs) commonly associated with conventional CTLA-4 blockade (74). The PRESERVE-004 Phase II trial (NCT04140526) evaluated gotistobart in combination with pembrolizumab in patients with advanced solid tumors, including a cohort of patients with PROC. In the PROC subset, the regimen demonstrated an objective response rate (ORR) of 25–27.6% and showed a manageable safety profile, marked by a lower incidence of high-grade irAEs compared to historical data with ipilimumab-based combinations. The disease control rate (DCR) was 58.6%. Treatment-related adverse events (TRAEs) of grade ≥3 occurred in 34.5% of patients, and only 6.9% experienced grade ≥3 immune-related adverse events (irAEs), representing a safety profile that compares favorably with the toxicity historically associated with conventional CTLA-4 inhibitors. These results suggest that this next-generation CTLA-4 inhibitor may offer an improved therapeutic window for combination immunotherapy in PROC (75). Beyond antibody-based approaches, cell therapies are also being explored in PROC, albeit at an earlier stage. Despite the inherent challenges of applying chimeric antigen receptor T-cell (CAR-T) therapy to solid tumors, research into its use for ovarian cancer persists (76). Tumor-infiltrating lymphocyte (TIL) therapy has demonstrated efficacy in treating melanoma, and its potential to induce sustained remission has been demonstrated in early studies in ovarian cancer (77). Furthermore, chimeric antigen receptor-engineered macrophages (CAR-M) and natural killer cells (CAR-NK) have emerged as a promising area of research due to their improved tumor infiltration and distinct toxicity profiles (78). Although early monotherapy with 4-1BB agonists such as urelumab was associated with limited efficacy attributable to dose-limiting toxicities, combination strategies are revitalizing interest in their therapeutic potential. Preclinical and early-phase clinical studies suggest that combining 4-1BB agonists with PD-1 blockade may enhance anti-tumor immunity in ovarian cancer (79). A phase I trial of the OX40 agonist GSK3174998 in combination with pembrolizumab (NCT02528357) has been completed, demonstrating target engagement and an acceptable safety profile in patients with advanced solid tumors, though clinical activity was limited (80). Future validation in larger, randomized phase III trials is warranted to confirm the promising early results observed with gotistobart and to definitively establish its role in the PROC treatment landscape.

Emerging strategies such as bispecific antibodies, agonist antibodies, and novel CTLA-4 inhibitors provide innovative tools to overcome the immune resistance in PROC, but most of their clinical evidence is still at an early stage, and the long-term profile of efficacy and safety is not yet fully clear. Among next-generation agents, the pH-sensitive anti-CTLA-4 antibody gotistobart (BNT316/ONC 392) combined with pembrolizumab has been evaluated in the PROC cohort of the phase II PRESERVE 004 trial, showing an ORR of 25–27.6%, a DCR of 58.6%, and a favorable safety profile (with grade ≥ 3 irAEs occurring in only 6.9% of patients) (76). The PD-1/CTLA-4 bispecific antibody cadonilimab (AK104) has been approved in China for recurrent cervical cancer (ORR 33.0%) and is being investigated in PROC (NCT05430906) (71, 73). In contrast, a randomized phase II trial evaluating conventional CTLA-4/PD-1 dual blockade with tremelimumab plus durvalumab demonstrated limited efficacy in PROC (ORR 8.7%, median PFS <2 months) (13). The OX40 agonist GSK3174998 combined with pembrolizumab demonstrated acceptable safety but limited clinical activity in advanced solid tumors (69). These data suggest that refining the pharmacology of existing targets may widen the therapeutic window, while bispecific T-cell engagers and agonist antibodies remain investigational and require further optimization of dosing, scheduling, and combination partners. In contrast to relatively mature combination regimens (such as immunotherapy combined with anti-angiogenic therapy or chemotherapy), the advantage of these novel strategies lies in their more sophisticated mechanisms of action (for example, bispecific antibodies can facilitate the formation of immune synapses, while pH-sensitive CTLA-4 antibodies can selectively modulate Tregs), with the goal of optimizing the therapeutic window. However, a shared limitation is the paucity of clinical data, with optimal dosing, administration schedules, and predictive biomarkers still requiring exploration through large-scale trials. The development of these strategies will depend on innovative clinical trial designs (such as umbrella trials and adaptive platform trials) to efficiently validate their value and requires integration with in-depth translational research to elucidate the response mechanisms in PROC patients stratified by distinct immune microenvironment subtypes, thereby achieving a reliable translation from “mechanistic innovation” to “clinical benefit”. As summarized in **Table 3**, the various immunotherapy combination strategies discussed above—including those with anti-angiogenics, PARP inhibitors, chemotherapy, ADCs, and novel agents—each exhibit distinct mechanisms of action, clinical efficacy profiles, and limitations. This comparative overview underscores the fact that no single strategy is universally superior; rather, the selection should be guided by the specific tumor microenvironmental context, biomarker status, and patient tolerability.

## 5 Critical analysis of negative phase III trials

While multiple phase II studies have shown promising efficacy of immunotherapy combinations in PROC, the transition to phase III has been marked by numerous setbacks. A systematic analysis of these negative trials is essential to inform future trial design and prevent the replication of these unsuccessful approaches. This section critically evaluates major phase III trials that failed to meet their primary endpoints, focusing on three combination strategies: ICI plus PARP inhibitors, ICI plus anti-angiogenic agents, and ICI plus chemotherapy.

### 5.1 ICI plus PARP inhibitors: the PFS – OS discordance

The DUO-O trial (NCT03737643) evaluated durvalumab added to carboplatin/paclitaxel/bevacizumab followed by durvalumab/bevacizumab with or without olaparib maintenance in patients with newly diagnosed non-tBRCAm advanced ovarian cancer (81). The primary analysis demonstrated a statistically significant PFS benefit for the durvalumab + olaparib arm versus control in the non-tBRCAm HRD-positive (HR 0.49, 95% CI 0.34–0.69) and ITT populations (HR 0.63, 95% CI 0.52–0.76; p < 0.0001). However, at final PFS and interim OS analysis, the OS HR was 0.95 (95% CI 0.76–1.20; p = 0.68) in the ITT population, indicating no OS benefit despite PFS improvement. Grade ≥ 3 adverse events were higher in the olaparib arm (57% vs 49%) (81). The FIRST/ENGOT-OV44 trial (NCT03602859) evaluated dostarlimab added to first-line platinum-based chemotherapy plus niraparib maintenance with or without bevacizumab in patients with newly diagnosed advanced ovarian cancer (42). The trial showed a statistically significant but clinically modest PFS improvement with dostarlimab (median 20.6 vs 19.2 months; HR 0.85, 95% CI 0.73–0.99; p = 0.0351). OS was not significantly different (median 44.4 vs 45.4 months; HR 1.01, 95% CI 0.86–1.19; p = 0.9060) (42).

Critical interpretation: These results highlight a recurring phenomenon — a statistically significant but clinically marginal PFS benefit that fails to translate into OS improvement. Several factors may explain this discordance: (1) post-progression therapy confounding — patients in the control arm may have received effective salvage therapies (including PARP inhibitors or ICIs) following disease progression, diluting any OS benefit; (2) overlapping toxicity leading to dose reductions or early discontinuations, undermining the intended synergistic effect; (3) insufficient biomarker enrichment — both trials included unselected ITT populations, whereas preclinical data suggest that HRD-positive and STING-pathway-activated tumors derive greater benefit; and (4) the immunomodulatory effects of PARP inhibitors may be less impactful in the first-line setting where baseline immune recognition is lower compared to heavily pretreated recurrent disease in which phase II signal was observed.

### 5.2 ICI plus anti-angiogenic agents: failure in first line and recurrent settings

In the first-line setting, the phase III IMagyn050/GOG 3015/ENGOT OV39 trial (NCT03038100) evaluated atezolizumab in combination with carboplatin/paclitaxel/bevacizumab in 1301 patients with newly diagnosed stage III/IV ovarian cancer (33). The addition of atezolizumab failed to significantly prolong PFS in the ITT population (median 19.5 vs 18.4 months; HR 0.92, 95% CI 0.79–1.07; p = 0.2785) (33). In the PROC setting, the JAVELIN Ovarian 200 trial (NCT02580058) randomly assigned 566 patients with platinum-resistant or -refractory ovarian cancer to avelumab monotherapy, avelumab + PLD, or PLD alone (10). Neither avelumab monotherapy (median PFS 1.9 months; HR 1.68) nor avelumab + PLD (median PFS 3.7 months; HR 0.78) showed a statistically significant improvement in PFS or OS versus PLD alone (10). The recently reported AGO OVAR 2.29/ENGOT OV34 trial (NCT03353831) evaluated atezolizumab combined with bevacizumab and non-platinum chemotherapy (weekly paclitaxel or PLD) in 574 patients with recurrent ovarian cancer ineligible for platinum (82). The final analysis showed no significant improvement in OS (median 14.2 vs 13.0 months; HR 0.83, 95% CI 0.68–1.01; p = 0.06) or PFS. Importantly, 72% of patients were pretreated with bevacizumab, and 36% had received three prior lines of therapy, indicating a heavily pretreated population with potentially exhausted immune effector function (82).

Critical interpretation: These results highlight a recurring phenomenon — a statistically significant but clinically marginal PFS benefit that fails to translate into OS improvement. Several factors may explain this discordance: (1) confounding by post-progression therapies — patients in the control arm may have received effective salvage therapies (including PARP inhibitors or ICIs) following disease progression, diluting any OS benefit; (2) overlapping toxicities leading to dose reductions or early discontinuations, undermining the intended synergistic effect; (3) insufficient biomarker enrichment — both trials included unselected ITT populations, whereas preclinical data suggest that HRD-positive and STING pathway-activated tumors derive greater benefit; and (4) the immunomodulatory effects of PARP inhibitors may be less impactful in the first-line setting where baseline immune recognition is lower compared to heavily pretreated recurrent disease in which phase II signal was observed.

### 5.3 Other failed or abandoned strategies

The phase III ARTISTRY 7 trial (NCT05092360) evaluated the novel IL-2 fusion protein nemvaleukin alfa in combination with pembrolizumab in patients with PROC (6). At the prespecified interim analysis, the combination did not achieve a statistically significant improvement in OS (median 10.1 vs 9.8 months; HR 0.98), and development for PROC was subsequently discontinued (6). In the ICI plus chemotherapy domain, the KEYNOTE B96 trial (ENGOT ov65, NCT05116189) stands as the first phase III trial to report a positive OS benefit for an ICI-containing regimen in PROC (83). Pembrolizumab added to paclitaxel with or without bevacizumab demonstrated a 4.2-month improvement in median OS compared with placebo in PD-L1 ≥ 1 patient (83). However, this positive result requires contextual interpretation: the OS benefit was observed only in PD-L1–selected patients, and the control arm’s modest survival underscores the urgent need for effective therapies in this population.

### 5.4 Cross-trial synthesis: lessons learned from negative phase III trials

A systematic review of phase III trials in PROC by Martorana et al. (2025) analyzed studies in platinum-resistant ovarian cancer to understand their poor outcomes and guide future trials (84). The review concluded that negative outcomes likely stem from three overarching factors: inconsistent definitions of platinum resistance across trials, inadequate control arm benchmarks that fail to reflect contemporary standards of care, and suboptimal use of predictive biomarkers for patient selection (84). From a biological perspective, the failure of these trials underscores that the PROC tumor microenvironment is not merely”immuno cold” but is characterized by multilayered, redundant immunosuppressive mechanisms that single-modality ICI combinations cannot easily overcome. Tumor heterogeneity, low tumor-infiltrating lymphocyte density, and the complex interplay between metabolic stress (hypoxia, acidosis) and immune escape pathways collectively create a hostile microenvironment that resists even rationally designed combination therapies. For future trials, these lessons mandate a shift from “one-size-fits-all” approaches to biomarker-driven enrichment strategies, as well as the incorporation of adaptive trial designs that allow for early termination of ineffective arms. Furthermore, given the high rate of discordance between PFS and OS in these trials, OS should remain the primary efficacy benchmark in future PROC registration-directed studies, with PFS interpreted cautiously as a surrogate endpoint.

## 6 Challenges and future directions

*Biomarker exploration.* Identification of reliable predictive biomarkers is key to improving the efficacy and cost-effectiveness of PROC immunotherapy. Existing markers such as PD-L1 (detected by immunohistochemistry, IHC), TMB (detected by next-generation sequencing, NGS panel/WES) and MSI-H/dMMR (detected by IHC/PCR/NGS) have limited predictive value in ovarian cancer (85). PD-L1 expression exhibits low and heterogeneous positivity rates in ovarian cancer. Ovarian cancer typically exhibits intermediate TMB, and the incidence of MSI-H is low (<2%) (86). Therefore, there is an urgent need to develop new biomarker strategies. Regarding TILs, the density of CD8+ T cells within the tumor core or immune fringe, quantified as the Immunoscore, represents a potential predictor (87). Additionally, immunogenetic signatures, derived from immune-related gene expression profiles (e.g., the IFN-γ signature and T-cell inflammatory gene profiles) in tumor tissues via NanoString or RNA-seq technologies, may provide a more comprehensive reflection of the tumor’s immune status (88). Furthermore, HRD-positive tumors may be more sensitive to combination immunotherapy involving PARP inhibitors due to their higher genomic instability and immunogenicity (85). Finally, accumulating evidence suggests that gut microbiota composition correlates with the efficacy of ICIs (89). Modulating the microbiota (e.g., via fecal microbiota transplantation or probiotics) represents a promising novel adjunctive strategy to enhance therapeutic efficacy (90).

Future research could explore developing integrated biomarker models that combine the aforementioned factors (such as TIL density, GEP, and HRD status) or incorporate dynamic monitoring of ctDNA (91) though such approaches require prospective validation in PROC. For instance, recent studies leveraging integrated multi-omics (e.g., combining whole-exome sequencing, RNA sequencing, and multiplex immunofluorescence) are defining specific immune-evasion subtypes and novel therapeutic vulnerabilities in ovarian cancer, providing a blueprint for such predictive models (92). As reviewed by Hatamikia et al., such integrative approaches systematically correlate genomic, transcriptomic, proteomic, and radiomic data to identify patterns that are undetectable by single-omics analyses. This framework aims to uncover distinct molecular subtypes, predict treatment response, and identify novel biomarker combinations, thereby paving the way for more precise patient stratification in PROC (92). Multi-omics analysis integrating genomics, transcriptomics, proteomics, and immunomics has been proposed as a promising approach for developing predictive models (93, 94) but its role in PROC remains to be defined.

Overcoming resistance mechanisms. Primary and acquired resistance represent critical hurdles in immunotherapy. Characterized by their complexity and diversity, these mechanisms are elucidated in this section.

IFN-γ pathway defects. Mutations or epigenetic silencing in genes of the IFN-γ signaling pathway (such as Janus kinase 1/2 (JAK1/2), signal transducer and activator of transcription 1 (STAT1), and interferon regulatory factor 1 (IRF1)) may result in defective antigen presentation and resistance to ICIs (95).

Alternative immune checkpoint upregulation. Under selective pressure from anti-PD-1/PD-L1 therapy, tumors may upregulate other immune checkpoints such as T-cell immunoglobulin and mucin domain-containing protein 3 (TIM-3), LAG-3, and TIGIT as compensatory immune escape mechanisms (96).

Tumor heterogeneity and clonal evolution. Distinct subclones coexist within the tumor, and treatment may select against clones exhibiting low immunogenicity or possessing resistance mechanisms, thereby driving disease progression (97).

Irreversible immunosuppressive microenvironment. Fibrotic mesenchyme, characterized by an abundance of immunosuppressive cells that are resistant to clearance, may result in irreversible ‘desertification’ or ‘immune exclusion’ of the TME (98). Strategies to overcome this must be multi-pronged and include the development of inhibitors against novel targets (TIM-3, LAG-3, and TIGIT) (99); co-targeting of metabolic pathways (IDO and CD73/adenosine) (100); and the exploration of repurposing existing metabolic-modulating drugs (such as metformin or statins). While initially developed for diabetes and hypercholesterolemia, respectively, these agents demonstrate pleiotropic immunomodulatory effects relevant to cancer therapy. Metformin, an AMP-activated protein kinase (AMPK) activator, has been shown to reduce both the accumulation and the suppressive function of myeloid-derived suppressor cells (MDSCs) and regulatory T cells (Tregs) within the TME, while simultaneously enhancing the cytotoxicity of CD8^+^ T cells and natural killer (NK) cells (101, 102). These effects collectively contribute to reversing the immunosuppressive TME and may overcome primary or acquired resistance to ICIs. The therapeutic potential of combining these widely available, cost-effective agents with immunotherapy in PROC warrants further clinical investigation (101). Future strategies to overcome resistance may include the repurposing of existing metabolic-modulating drugs (such as metformin or statins), given their increasingly recognized immunomodulatory potential (101); the remodeling of the TME via the administration of cytokines (e.g., IL-2, IL-12) or antagonists (e.g., anti-TGF-β) (22, 100); and the application of epigenetic drugs to regulate immune-related gene expression (100). however, direct evidence in PROC is limited, and most suggestions derive from preclinical or non-ovarian cancer models.

Optimizing clinical trial design. Umbrella and basket trials such as the BRIGHT study enroll patients into different sub-studies based on distinct biomarkers and test multiple targeted or immunotherapy regimens simultaneously to facilitate the efficient implementation of precision medicine (103).

Adaptive design allows the protocol to be adjusted according to the results of the interim analysis of the trial, such as discontinuing ineffective arms, adjusting the randomization ratio, or adding a new arm, to improve the efficiency of research and development (104). A master protocol is an overarching framework with multiple sub-studies that allows for the ongoing inclusion of new drugs or biomarkers to be tested. In addition, as PROC treatments are predominantly palliative, maintaining or improving quality of life is critical; therefore, patient-reported outcomes should serve as key secondary endpoints (105). Finally, real-world data can serve as an external control or be used for generating hypotheses to complement randomized controlled trials (106). Adaptive design allows for protocol adjustments based on accumulating data during the trial (such as modifying enrollment criteria, adjusting doses, or discontinuing ineffective arms) and is key to efficiently testing complex combination strategies and accelerating drug development. Future trial designs for PROC must integrate insights derived from the outcomes of key completed studies (84). **Table 4** synthesizes essential details from pivotal clinical trials evaluating immunotherapy in PROC, including their design, patient populations, and primary outcomes. Analyzing these results underscores the importance of biomarker-driven patient selection and adaptive trial designs to improve success rates.

**Table 4:** Summary of key clinical trials of PROC immunotherapy combination.

Trial name (phase)	Treatment plan	Patient population	Primary endpoint	Results, ORR/mPFS/mOS
KEYNOTE-100 (II) (9)	Pembrolizumab monotherapy	Late recurrent OC	ORR	8% (CPS ≥1); 9.7% (CPS ≥10)
JAVELIN Ovarian 200 (III) (10)	Avelumab ± PLD	Platinum-resistant/refractory ovarian cancer	PFS, OS	Not significantly improved
TOPACIO/KEYNOTE-162 (II) (44)	Niraparib + Pembrolizumab	PROC (not limited to BRCA)	ORR	18%; DCR, 65%
China basket research (II) (35, 37)	Carelli Zhu Famitinib	PROC	ORR	24.3%; mPFS, 4.1 months; mOS, 18.9 months
SORAYA (III) (35)	mirvetuximab monotherapy	High FRα expression PROC	ORR	42.3%
InnovaTV 205 (I/II) (64, 65)	Tisotumab vedotin ± Palbociclib	Recurrent OC (including PROC)	Safety, ORR	TV monotherapy ORR 22% (PROC)

Clinical translation: from biological rationale to patient benefit. Based on the evidence synthesized in this review, the following priorities are suggested for future clinical development, though each requires prospective validation. The ultimate goal of mechanistic and clinical research in PROC is to translate combination immunotherapy strategies into clinically meaningful benefits in patient outcomes (**Table 5**). To accelerate this translation, the following clinical development priorities are proposed:

**Table 5:** Summary of efficacy, safety, and key limitations of immunotherapy combination strategies in platinum-resistant ovarian cancer (PROC).

Strategy	Representative study	Key biomarkers	ORR	Median PFS	Grade ≥3 TRAEs	Key limitations
ICI + anti-angiogenic	Camrelizumab + famitinib (phase II) (29)	None	24.3%	4.1 mo	81.1%	High toxicity burden; no phase III validation
ICI + anti-angiogenic	Lenvatinib + pembrolizumab (phase II, LEAP-005) (111)	None	26% (INV)/35% (BICR)	6.2 mo	77%	Fourth-line setting; single-arm design
ICI + PARPi	Niraparib + pembrolizumab (phase I/II, TOPACIO) (39)	BRCA-independent (ORR 42% in BRCA-mut, 11% in wild-type)	25%	NR	~69% (grade ≥3)	Phase II only; no phase III OS benefit
ICI + PARPi	Olaparib + durvalumab (phase II, MEDIOLA) (40)	gBRCAm enriched	34%	5.5 mo	~50%	Small sample size (n=32); PSROC + PROC mixed
ICI + chemotherapy	Pembrolizumab + paclitaxel ± bevacizumab (phase III, KEYNOTE-B96) (18, 112, 113)	PD-L1 CPS ≥1 (OS benefit 18.2 vs 14.0 mo, HR 0.76)	NR	8.3 mo (ITT) vs 6.4 mo (control); HR 0.70	Consistent with known profiles	OS benefit only in PD-L1-selected patients at interim analysis; final OS data awaited for all-comers
ADC monotherapy	Mirvetuximab soravtansine (phase III, SORAYA) (58)	FRα high (TPS ≥75%)	31.7%	4.1 mo	39%	Biomarker restricted; single-agent limitation
ADC combination	Mirvetuximab + pembrolizumab (phase III ongoing, GLORIOSA)	FRα high	TBD	TBD	TBD	Pending full results
Dual ICI (novel)	Gotistobart + pembrolizumab (phase II, PRESERVE-004) (19) (20)	None	25.0–27.6%	Pending	31.0–35.7% (grade ≥3 TRAEs)	Phase II only; phase III confirmation pending
ADC (MSLN-targeting)	Anetumab ravtansine + bevacizumab (phase II) (114)	MSLN positivity (88%)	21% (ARB)	5.3 mo	18% (anemia)	

1. Biomarker-driven enrichment for phase III trials.

The discordant results between phase II and phase III trials underscore the necessity of prospective biomarker selection. Future pivotal trials should restrict enrollment to molecularly defined subgroups, such as HRD-positive, STING-pathway-high, or PD-L1-positive (CPS ≥ 1) populations, rather than unselected PROC patients.

2. Standardization of combination regimens and toxicity management.

The optimal dose, schedule, and sequence of combination therapies are still largely empirical. For ICI–anti-angiogenic combinations, low-dose lenvatinib (14 mg/day) plus toripalimab has shown a favorable safety profile and should be further validated (107). For ICI–PARPi combinations, intermittent dosing of PARP inhibitors may reduce cumulative myelotoxicity while preserving immunomodulatory effects. Multidisciplinary toxicity management protocols are essential for ADCs and novel immunotherapies.

3. Integration of patient-reported outcomes (PROs) as co-primary endpoints.

Given that PROC treatments are predominantly palliative, regulatory agencies increasingly require PRO data alongside traditional efficacy endpoints. Future trials should utilize validated PRO instruments to capture quality of life, symptom burden, and functional status.

4. Adaptive platform trials for efficient regimen screening.

Given the multitude of potential combination strategies, traditional two-arm phase III trials are inefficient. Master protocol designs that allow for dynamic addition and removal of arms based on interim biomarker data should be adopted. The BRIGHT study (42) serves as a model for biomarker-driven, multi-arm adaptive designs in PROC.

5. Real-world evidence (RWE) to complement randomized trials.

High-quality real-world data from registries and electronic health records can provide external validity, assess generalizability, and generate hypotheses for rare subgroups. RWE should be harmonized using common data models to enable pooled analyses across institutions.

In summary, bridging the gap between biological insight and clinical practice in PROC requires a coordinated effort encompassing biomarker validation, optimized trial designs, toxicity management guidelines, and patient-centric endpoints. Only through such a comprehensive translational framework will immunotherapy combinations deliver meaningful benefits in both survival and quality of life to patients with this challenging disease.

Security management. One of the core challenges of combination immunotherapy is the overlapping toxicities or the emergence of new adverse event profiles. For example, combining ICIs with anti-angiogenic TKIs not only leads to immune-related hepatitis and pneumonitis but also notably increases the risk of hypertension, proteinuria and hand-foot syndrome; combining ICIs with PARP inhibitors necessitates careful monitoring for cumulative bone marrow suppression and severe irAEs. In addition, the inclusion of ADCs introduces unique management challenges such as ocular toxicity and neuropathy. Therefore, clinical decisions must carefully weigh therapeutic benefits against toxicity risks, highlighting the critical importance of developing predictive toxicity biomarkers and establishing multidisciplinary toxicity management teams. Detailed incidence rates and severity data for these toxicities are presented in the respective clinical evidence sections above (e.g., Sections 4.1-4.5), as reported in the cited primary clinical studies. Immunological combination therapy is inherently associated with the risk of increased toxicity, and irAEs can involve any organ system (108). The toxicity profile of combination therapies (such as ICI + ICI and ICI + TKI) may be distinct, additive, or overlapping, manifesting as hepatitis, colitis, pneumonia, and endocrine disorders (107). Effective safety management strategies include: i) Pre-treatment risk assessment by obtaining a detailed history, conducting a baseline examination, and identifying high-risk patients; ii) prophylactic measures such as pretreatment with glucocorticoids to reduce the risk of infusion reactions and certain irAEs; iii) patient and healthcare provider education, which encompasses instructing patients on the early recognition and reporting of irAE symptoms; iv) implementation of timely and standardized interventions based on Common Terminology Criteria for Adverse Events (CTCAE) grading upon the onset of irAEs, typically involving drug suspension and the administration of corticosteroids or other immunosuppressive agents (109); and v) multidisciplinary team collaboration, as the management of complex irAEs (such as myocarditis and neurological toxicity) requires experts in oncology, rheumatology, endocrinology, gastroenterology, dermatology, and other disciplines (110).

The methodological quality assessment identified that while most RCTs exhibited low to moderate risk of bias, several single-arm phase II studies were rated as having moderate risk of bias due to the absence of comparator arms, potential confounding, and incomplete reporting of baseline characteristics. Notably, the overall risk-of-bias rating was “low” for key trials with rigorous design, whereas a rating of “some concerns” or “moderate” was assigned to studies with open-label designs or *post-hoc* subgroup analyses. Consequently, the findings from the higher-quality trials lend stronger support to the main conclusions of this review. Conversely, findings from studies with “some concerns” or “moderate” risk of bias should be interpreted as exploratory and hypothesis-generating, rather than as definitive evidence. These considerations were explicitly accounted for in the narrative synthesis presented in Section 3, where findings from lower-quality studies are discussed with appropriate caution and not presented as conclusive support for the efficacy of a given regimen. To translate these findings into practice, we propose several recommendations. For ICI anti angiogenic combinations, baseline blood pressure should be controlled (<140/90 mmHg) with home monitoring twice daily during the first 4 weeks; low dose lenvatinib (14 mg/day) is preferred over higher doses to reduce toxicity (107). For ICI PARPi combinations, a baseline complete blood count with weekly monitoring for the first 8 weeks is advised; intermittent PARPi dosing can manage grade ≥ 3 cytopenias. For ADC containing regimens, baseline and regular (every 6 weeks) ophthalmologic examinations are mandatory to mitigate ocular toxicity. Finally, all centers should establish a rapid access multidisciplinary team and provide patients with a 24/7 hotline and an irAE action card listing symptoms requiring immediate reporting. Regarding real-world applicability, despite promising trial results, the cost and accessibility of novel immunotherapy combinations remain major barriers. ADCs and next generation ICIs are expensive and largely confined to academic centers. Companion biomarker testing (FRα, HRD, PD L1) adds further complexity. In resource limited settings, low dose lenvatinib plus generic ICIs may represent a more pragmatic option. Real world evidence and cost effectiveness studies are urgently needed to guide equitable implementation.

Cost, Accessibility, and Real-World Applicability

Although the above-mentioned immunotherapy combinations have shown certain efficacy in clinical trials, their clinical implementation faces significant economic and practical barriers.

High cost: ADC drugs cost approximately $15,000–$30,000 per cycle, immune checkpoint inhibitors cost about $10,000–$15,000 per dose, and PARP inhibitors exceed $10,000 per month. Most combination regimens cost over $200,000 per year, far beyond the affordability of ordinary patients.

Uneven accessibility: These new drugs are mainly concentrated in large cancer centers and developed regions. Companion diagnostic testing requires a specialized pathology platform, which is difficult for primary hospitals to carry out. Low- and middle-income countries still primarily rely on traditional single-agent chemotherapy.

Real-world applicability recommendations: In resource-limited settings, priority can be given to regimens such as low-dose lenvatinib (14 mg/day) combined with domestic PD-1 inhibitors; or to regimens that have shown positive results, such as pembrolizumab combined with paclitaxel. ADCs should be limited to patients with high FRα expression and are not suitable for widespread use.

Economic evaluation pending: Currently, there is no formal cost-effectiveness analysis for PROC immunotherapy combinations. Based on an estimated overall survival benefit of 4.2 months, the incremental cost-effectiveness ratio may exceed $200,000 per quality-adjusted life year, higher than the conventional willingness-to-pay threshold. Future phase III trials need to concurrently conduct health economic evaluations.

In summary, when selecting medications, clinicians need to consider efficacy, toxicity, and the patient’s economic conditions comprehensively, avoiding blindly pursuing new combination drugs.

## 7 Conclusion

For ICI PARPi combinations, a baseline complete blood count with weekly monitoring for the first 8 weeks is advised; intermittent PARPi dosing can manage grade ≥ 3 cytopenias. For ADC containing regimens, baseline and regular (every 6 weeks) ophthalmologic examinations are mandatory to mitigate ocular toxicity. Finally, all centers should establish a rapid access multidisciplinary team and provide patients with a 24/7 hotline and an irAE action card listing symptoms requiring immediate reporting. Regarding real-world applicability, despite promising trial results, the cost and accessibility of novel immunotherapy combinations remain major barriers. ADCs and next generation ICIs are expensive and largely confined to academic centers. Companion biomarker testing (FRα, HRD, PD L1) adds further complexity. In resource limited settings, low dose lenvatinib plus generic ICIs may represent a more pragmatic option. Real world evidence and cost effectiveness studies are urgently needed to guide equitable implementation.

## 8 Limitations of the review

Despite the systematic and comprehensive approach adopted in this review, several inherent limitations must be considered when interpreting the findings. First, substantial clinical and methodological heterogeneity exists across the included studies, precluding a quantitative meta-analysis and necessitating a narrative synthesis. The patient populations exhibited considerable variation in terms of prior lines of therapy, definitions of platinum resistance, and the extent of pretreatment with agents such as bevacizumab or PARP inhibitors. In addition to patient-level factors, differences in study designs—ranging from phase II single-arm trials to randomized phase III studies—along with variations in immunotherapy agents, combination partners, dosing schedules, and primary endpoints, limit the direct comparability of outcomes across strategies.

Second, the potential for publication bias cannot be ruled out. Peer-reviewed journals are more likely to publish studies with positive or promising results, while negative phase III trials are often subject to reporting delays or presented with less comprehensive data reporting. This may lead to an overestimation of the true effect size of certain combination regimens. Moreover, most of the included phase II studies had modest sample sizes and were not powered for definitive efficacy comparisons, increasing the risk of false-positive findings.

Third, the available evidence is limited by the heterogeneity in methodological quality of the primary studies. As detailed in **Table 2**, while key randomized trials exhibited low risk of bias, several single-arm phase II studies were rated as having moderate risk of bias due to the absence of comparator arms, lack of blinding, incomplete reporting of baseline prognostic factors, or the reliance on *post-hoc* subgroup analyses. Findings from these studies should therefore be considered hypothesis-generating rather than practice-changing.

Ultimately, long-term safety and survival data remain immature for many emerging combination strategies, particularly those involving novel agents such as bispecific antibodies, pH-sensitive CTLA-4 inhibitors, and antibody-drug conjugates. The durability of responses and the cumulative toxicity profiles over extended follow-up remain to be fully elucidated. Consequently, the conclusions presented herein reflect the current state of evidence and are subject to revision as more mature data become available from ongoing and future phase III trials.
